# The *in vivo* mechanics of the magnetotactic backbone as revealed by correlative FLIM-FRET and STED microscopy

**DOI:** 10.1038/s41598-019-55804-5

**Published:** 2019-12-23

**Authors:** Erika Günther, André Klauß, Mauricio Toro-Nahuelpan, Dirk Schüler, Carsten Hille, Damien Faivre

**Affiliations:** 1 Max Planck Institute of Colloids and Interfaces, Department of Biomaterials, Am Mühlenberg 1, 14476 Potsdam, Germany; 2 Physical Chemistry, Institute of Chemistry, University of Potsdam, 14476 Potsdam, Germany; 3 Department of Microbiology, University Bayreuth, 95447 Bayreuth, Germany; 4 Present Address: HOLOEYE Photonics AG, 12489 Berlin, Germany; 5 Present Address: European Molecular Biology Laboratory, 69117 Heidelberg, Germany; 6 Present Address: Technical University of Applied Sciences Wildau, 15745 Wildau, Germany; 7 Aix Marseille Université, CEA, CNRS, BIAM, 13108 Saint Paul-Lez-Durance, France

**Keywords:** Biological fluorescence, Nanoscale biophysics, Chemical biology, Photochemistry, Physical chemistry, Biophysics, Chemical biology

## Abstract

Protein interaction and protein imaging strongly benefit from the advancements in time-resolved and superresolution fluorescence microscopic techniques. However, the techniques were typically applied separately and *ex vivo* because of technical challenges and the absence of suitable fluorescent protein pairs. Here, we show correlative *in vivo* fluorescence lifetime imaging microscopy Förster resonance energy transfer (FLIM-FRET) and stimulated emission depletion (STED) microscopy to unravel protein mechanics and structure in living cells. We use magnetotactic bacteria as a model system where two proteins, MamJ and MamK, are used to assemble magnetic particles called magnetosomes. The filament polymerizes out of MamK and the magnetosomes are connected via the linker MamJ. Our system reveals that bacterial filamentous structures are more fragile than the connection of biomineralized particles to this filament. More importantly, we anticipate the technique to find wide applicability for the study and quantification of biological processes in living cells and at high resolution.

## Introduction

Biological processes are very diverse and include for example the interaction between biomacromolecules^1^, their distribution in cells^2^, their structure^3^ and dynamics^4^. Due to its non-invasive approach, the high sensitivity and the availability of highly specific fluorophores, fluorescence microscopy has become one of the most powerful and versatile tool in life sciences to study the functional and structural aspects of these processes, both qualitatively and quantitatively^5^. The main drawback of conventional fluorescence microscopy is the limitation of the spatial resolution to about half a wavelength of the emission light. However, this diffraction barrier has been overcome by the development of superresolution fluorescence imaging techniques^6,7^. For example, in stimulated emission depletion (STED) microscopy, the theoretical spatial resolution scales approximately with the inverse square root of the depletion laser intensity. Thus, the spatial resolution of the probe is influenced by additional factors. Hence, novel, reliable calibration-free methods have been developed^8^. However, *in vivo* imaging remains challenging because of induced phototoxicity and photodamage resulting from the high STED laser intensities^9^. Therefore, due to a high number of excitation-depletion cycles, photobleaching limits extended imaging in living cells^10^. In addition, the long image acquisition times often hinder the detection of biological processes at their natural time scale^11^ Finally, STED is purely an imaging technique revealing structural information at high resolution.

Alternatively, Förster resonance energy transfer (FRET) allows for determining distances of interacting proteins at the nanometer scale (typically < 10 nm) *in vitro* and *in vivo*^12^. FRET measurements can be realized by monitoring fluorescence intensities or fluorescence decay times of donor and acceptor molecules, the latter being realized by combination with fluorescence lifetime imaging microscopy (FLIM-FRET). This approach only needs detection of donor decay times and additionally allows for distinguishing between interacting and non-interacting donor fractions, a drawback in intensity-based FRET measurements^13^. However, if both interacting proteins change their intracellular location during measurements or both are involved in configuration changes, then FLIM-FRET lacks direct information about structural and mechanistic insights. Therefore, combining STED microscopy with FLIM-FRET would resolve detailed structural as well as quantitative information about the molecules of interest. Combined FLIM-FRET/STED recordings have been reported recently^14–16^. There, organic fluorophores have been applied or recordings occurred in artificial systems (dsDNA fragments). Here, we develop a new FRET-pair based on fluorescent proteins expressed in a living biological system.

However, cellular mechanics is a typical example, for which microscopic techniques are used to decipher biological processes^17,18^. Due to the above-mentioned problems, the standard to address these problems *in vivo* often relies on alternative techniques possibly encompassing the internalization of magnetic beads and a poorly defined application of stress with expected bias in term of cell behavior^19^. To avoid these limitations, we have addressed the generic problem of cellular mechanics by using magnetotactic bacteria as a model system, in which magnetic forces can directly be employed to test the mechanics of biological players *in vivo* by X-ray diffraction^20^. These particular organisms, some of which are genetically modifiable^21^, indeed intracellularly mineralize magnetic nanoparticles^22^ termed magnetosomes and assemble them into a chain^23^. The magnetosome chain relies on the presence of a cytoskeletal filament, the actin-like MamK protein^24^, to which the magnetosomes are attached by the MamJ protein^25^. We reported that the magnetosome chain can withstand a force of *F* = 25 pN before breaking^20^, lower than forces reported in the actin-family^26^. Based on this difference and on conventional fluorescence microscopy, we suggested MamJ to be the weakest link in the assembly^20^. However, a direct experimental proof is missing.

Herein, we therefore integrate correlative FLIM-FRET and STED microscopy to image the filament structure and to resolve the MamJ/MamK interaction at nanometer scale. We show, how the combination of these high-end optical techniques reveals that contrary to the expectation, the bacterial filament is more fragile than its connection to the magnetosome membrane and finally comment on possible application of the technique in further fields.

## Results and Discussion

### FLIM-FRET demonstrates the interaction between MamJ and MamK within a magnetotactic bacterium

**Figure 1:**
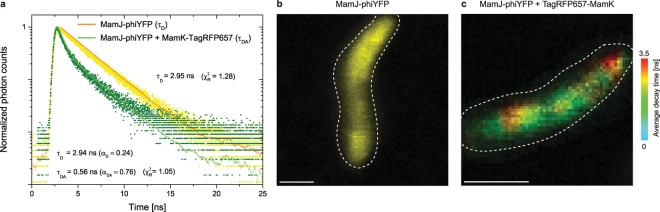
*In vivo* FLIM-FRET analysis in MSR-1 cells with MamJ-phiYFP and MamJ-phiYFP + TagRFP657-MamK. (**a**) Representative phiYFP (donor) fluorescence decay curves measured by time-correlated single-photon counting (TCSPC) in MSR-1 expressing either MamJ-phiYFP (yellow data points) or MamJ-phiYFP together with TagRFP657-MamK (green data points) translational fusions. For MamJ-phiYFP alone, the data was fitted to a single-exponential deconvolution fitting model (orange line), yielding the decay time τ_D_ = 2.95 ns. For MamJ-phiYFP in the presence of TagRFP657-MamK, the data was fitted to a bi-exponential deconvolution fitting model (green line), resulting in τ_D_ = 2.94 ns (fixed value, determined from n = 9, SI) and τ_DA_ = 0.56 ns. (**b**) FLIM images of MSR-1 with MamJ-phiYFP alone or (**c**) together with TagRFP657-MamK. The amplitude-weighted average decay time for phiYFP is shown in pseudo-color code, in which warmer colors represent the unaffected donor decay time and colder colors represent shorter decay times due to energy transfer between phiYFP and TagRFP657. Scale bar: 1 µm.

MamJ and MamK were shown to interact with the 2-hybrid assay^27^ and within a host organism^28^. To measure if the proteins were interacting directly within the magnetotactic bacteria, we choose the fluorescent proteins phiYFP and TagRFP657 as a new combination of FRET pair for FLIM-FRET measurements. We used the bacterial strain *Magnetospirillum gryphiswaldense* MSR-1, in which the translational fusion proteins MamJ-phiYFP (donor) and TagRFP657-MamK (acceptor) were expressed. Representative fluorescence decay curves of the donor phiYFP, either alone or in presence of the acceptor as TagRFP657-MamK (green), are shown (Fig. 1a).

By means of time-correlated single-photon counting (TCSPC), the fluorescence decay time of the donor protein phiYFP fused to MamJ was calculated as τ_D_ = 2.95 ns, which decreases significantly upon the presence of the acceptor protein TagRFP657 fused to MamK to τ_DA_ = 0.56 ns (details in SI). This observation indicates an energy transfer between the fluorophores, which in turn is a preliminary hint for a MamJ-MamK interaction. In addition, FLIM images demonstrate that phiYFP fused to MamJ exhibits a faster fluorescence decay behavior when the TagRFP657-MamK fusion is present (Fig. 1b,c, Supplementary Fig. S1). We firstly calculated the spectral overlap integral *J(λ*) = 1.34 × 10^15^ nm^4^ M^−1^ cm^−1^ and the Förster distance *R*_0_ = 4.6 nm, using Eqs. (2) and (3) (Methods, Supplementary Fig. S2) to characterize the phiYFP and TagRFP657 FRET pair. We determined the distance between the donor phiYFP and the acceptor TagRFP657 as *r* = 3.6 ± 0.2 nm (n = 72) (Eq. (4)) using the measured fluorescence decay times in individual cells. Thus, we demonstrated that phiYFP and TagRFP657 are suitable for FLIM-FRET measurements in living MSR-1 and that MamJ and MamK interact with each other, also in original magnetotactic bacteria.

### STED microscopy confirms that MamK forms filaments *in vivo*

**Figure 2:**
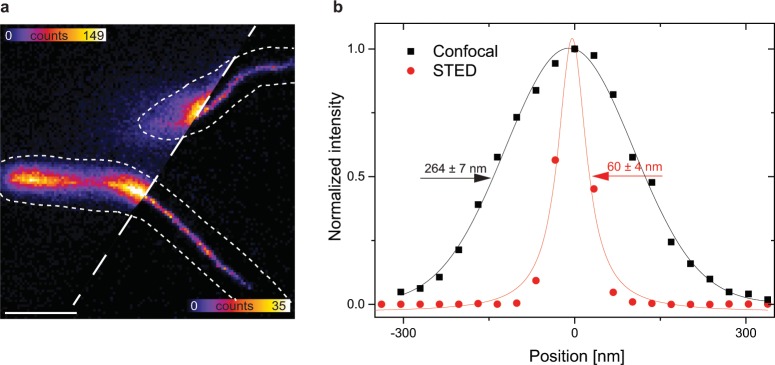
*In vivo* STED analysis in MSR-1 cells with TagRFP657-MamK. (**a**) Confocal and STED fluorescence image of MamK-TagRFP657 filaments in living MSR-1 along with an intensity line profile is plotted (dashed line) (**b**). Gaussian approximation for the exemplary confocal image in (**a**) provides a full-width at half maximum (FHWM) of 227 ± 6 nm (black) and Lorentzian fitting for STED image (**a**) results in a FHWM of 60 ± 4 nm (red). The error arises from fitting errors and not from multiple measurements. Line profiles represent normalized raw image data and fitting curves (fitted value ± standard error). Scale bar: 1 µm.

We have shown using photoactivated localization microscopy (PALM) that MamK-mCherry was forming filament *in vivo* with a resolution of about 150 nm^29^. However, this construct and technique do not allow the expected combine microscopy approach. Therefore, we next imaged only the acceptor TagRFP657 fused to MamK in a living MSR-1 cell that also display MamJ-phiYFP using both, conventional confocal and STED fluorescence microscopy (Fig. 2a,b). The apparent filament diameter of imaged filaments was enhanced by STED reaching a 4-fold reduction in filament diameter (about 60 vs. 240 nm, Supplementary Table S1). Since the fluorescence is subjected to photobleaching in STED imaging, we assessed the feasibility of consecutive image acquisition in the bacteria. Immediately after a first STED image acquisition, the fluorescence is bleached. However, after a regeneration period of 20 min, the fluorescence intensity of the filament is recovered such that a second STED image of similar quality can be recorded (SI, Supplementary Fig. S2).

### The combination of FLIM-FRET and STED microscopy as applied to the MamJ-MamK pair

**Figure 3:**
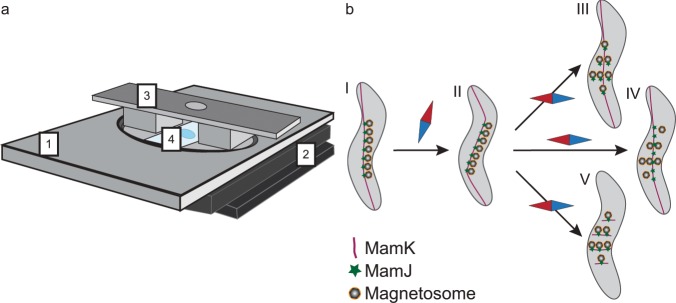
Setup and possible effects of rotating a magnetic field around a bacterium. (**a**) An aluminum plate (1) is placed on top of the stage of an inverted microscope (2) without contact. The magnet (3) can be freely rotated around the sample (4). (**b**) Possible scenarios of MamJ-MamK interaction caused by the external magnetic field. (I) Living bacteria are immobilized and aligned with an external magnetic field in an agarose matrix. (II) The magnetosome chain can initially follow the rotating field, up to a given threshold, where three possible effects are envisaged: (III) MamJ detaches from the filament so that MamJ and MamK are separated. A decreased FRET efficiency and an intact filament are expected. (IV) Alternatively, the magnetosomes are detached from the MamK filament, but MamJ and MamK are still connected. A high FRET efficiency similar to the starting case (I) and an intact MamK filament are expected. (V) Finally, the filament ruptures, but MamJ and MamK are still connected. An unaltered high FRET efficiency is assumed, but filament fragments are expected in STED images.

After establishing the feasibility of our microscopic approach, we tested the mechanical stability of the MamJ-MamK pair *in vivo* by combined FLIM-FRET and STED microscopy Fig. 3a. displays a sketch of the setup, which is equipped with an aluminum (non-magnetic) plate topped with a freely rotating magnet pair to test potential effects of rotating a magnetic field (applying a magnetic torque, strength of the magnetic field: 50 mT) around a magnetotactic bacterium (on the magnetosome chain) (Fig. 3b).

**Figure 4:**
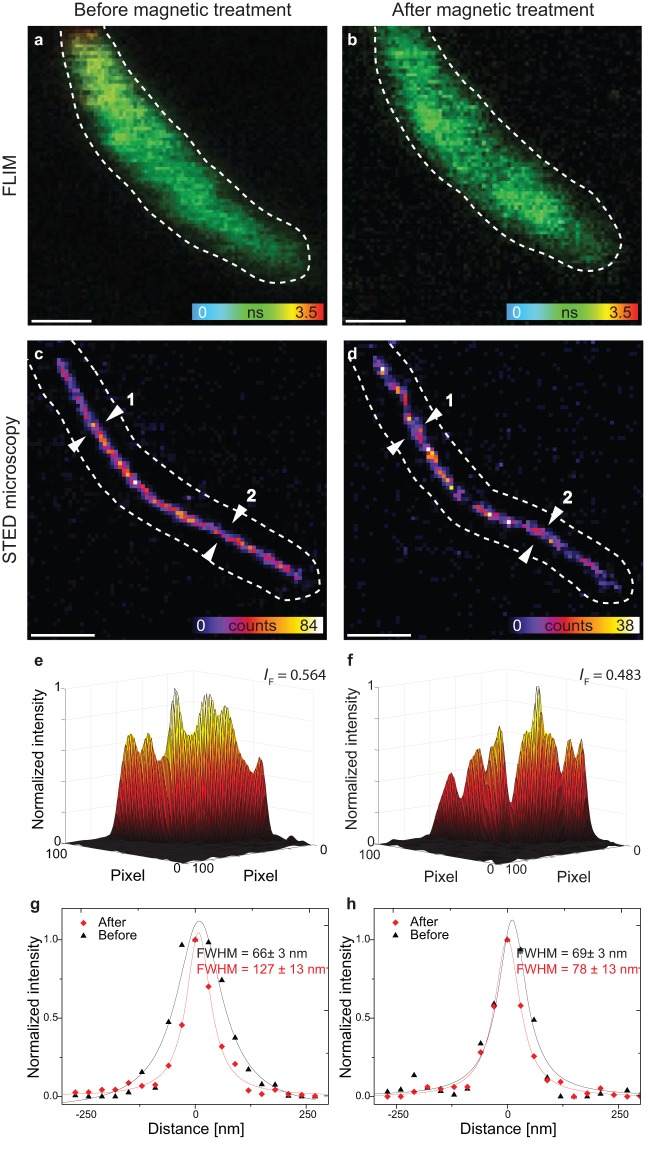
The *in vivo* FLIM-FRET and STED experiments on MSR-1 cells before and after application of a magnetic torque. FLIM images (**a,b**) and STED images (**c,d**) of MSR-1 cells with MamJ-phiYFP and TagRFP657-MamK before (**a,c**) and after (**b,d**) applying treatment (10 times rotation of two magnets up to 270°). (**e,f**) Corresponding 3D surface intensity plots of the STED images. We normalized the intensity and used a Gaussian filter. Normalized fluorescence intensities along the filament structures were calculated to *I*_F_ = 0.564 and *I*_F_ = 0.483, respectively (SI). (**g,h**) Intensity line profiles. Lorentzian approximation provides a full-width at half maximum (FHWM) at position 1 before magnetic treatment of 66 ± 3 nm and after magnetic treatment of 127 ± 3 nm. At position 2, the apparent filament diameter is 69 ± 3 nm before and 78 ± 13 nm afterwards. Intensity line profiles represent normalized raw image data and fitting curves (fitted value ± standard error). Scale bar: 1 µm.

FLIM images of an identical cell with MamJ-phiYFP accompanied by TagRFP657-MamK before and after application of a magnetic torque (Fig. 4a,b) and the corresponding STED images of the filament are shown (Fig. 4c,d). After the treatment, the donor-acceptor distance between MamJ and MamK (phiYFP and TagRFP657) was calculated from the FRET efficiencies as *r* = 3.4 ± 0.2 nm (n = 45), and remains unchanged (P > 0.05) compared to the measure performed before treatment (3.6 nm ± 0.2 nm), indicating that the MamJ-MamK interaction is unchanged (Supplementary Information, Supplementary Table S3). Decay times are thus similar before and after treatment (Fig. 4a,b).

In contrast, while a straight MamK filament is observed before treatment (Fig. 4c), structural defects are detected afterwards (Fig. 4d). Therefore, we quantified the TagRFP657 fluorescence intensity distributions from the STED images (Fig. 4e,f) by plotting the 3D surface intensity of cross sections at two random filament positions. After the treatment (Fig. 4f), sharp intensity changes are recorded, which, depending on the position, were absent or less pronounced before treatment (Fig. 4e). The filament diameter almost doubled at position 1 (from 66 nm to 127 nm), whereas it only increased by approx. 10 nm at positon 2, pointing towards non-uniform effects (Fig. 4g,h, additional discussion in SI).

As MamK filaments are known to be constructed from bundles of single protein units that polymerize^30^, this result can reproduce patterns observed at more fragile nods *vs*. more stable central segments of the helical structure builds from the protein. In order to quantify these changes in the MamK filament organization, we integrated the TagRFP657 fluorescence intensity along the filament (Supplementary Table S4). The same line with a width of 7 pixels was drawn along the ridge of filaments before and after magnetic treatment. The averaged TagRFP657 fluorescence intensities of pixels along the line were plotted against the length, thereby normalizing to the maximal intensity (SI). The area under the curve was determined by integration. Supplementary Table S4 summarizes the calculated area of 10 filaments before and after magnetic treatment. A normalized fluorescence intensity of *I*_F_ = 1.0 would correspond to maximum fluorescence intensities in all pixels along the whole structure reflecting best filament integrity. The mean and standard deviation of *I*_F_ of 10 MamK filaments (2 independent experiments) was calculated. Before application of a magnetic torque *I*_F_ = 0.457 ± 0.083 and decreases significantly to 0.374 ± 0.086 (n = 10) afterwards (paired *t*-test, P = 0.0139) (see also Supplementary Table S4). In contrast, the calculated area of the filaments before (*I*_F_ = 0.477 ± 0.068, n = 10) and after a 20 minute regeneration period (*I*_F_ = 0.436 ± 0.062, n = 10) are not significant different (paired *t*-test, P = 0.0658, Supplementary Table S2). Overall, these results indicate a loss of integrity of the MamK filament (Supplementary Table S4, Figs. S4 and S5). However, the *in vivo* STED images, do not constantly exhibit clear pattern of broken chains as those observed in chemically fixed cells using transmission electron microscopy (TEM)^20^. Since our experiments are performed in living cells, cellular processes are not inhibited. The formation of MamK filament in MSR-1 was recently shown to be dynamic, suggesting that MamK experience treadmilling^31^. The MamK treadmilling growth speed is 310–340 nm/min^31^. At least 50 s passes from magnetic treatment until the STED image is recorded (Supplementary Fig. S5). During this time *de novo* synthesized MamK could potentially grow explaining the recorded patterns since the *de novo* synthesis of MamK may be faster than the temporal resolution of STED image acquisition.

### Chemically fixed cells display broken MamK filaments and magnetosome chains

**Figure 5:**
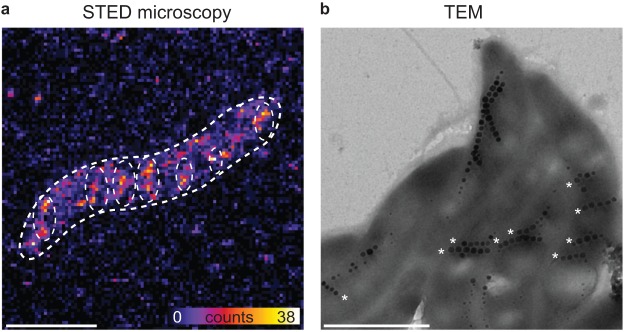
Filament and chain fragments of chemically fixed cells after magnetic treatment. STED image (**a**) and TEM image (**b**) of chemically fixed MSR-1 cells. Scale bar: 1 µm.

In order to assess the origin of these non-uniform effects, we chemically fixed the bacteria right after the application of the magnetic torque to freeze their internal structure and imaged them by both by STED microscopy and TEM. STED images reveal the absence of any cell-spanning, continuous MamK filament, which instead appear to be fragmented into many short filaments (Fig. 5a, Supplementary Fig. S6). The fragmented MamK filament pieces, highlighted in Fig. 5a (the image without highlights is shown in Supplementary Fig. S7) cross the cell along its short axis, contrary to the typical continuous MamK filament localization along the long cell axis. The TEM micrograph in Fig. 5b shows multiple MSR-1 cells. Here, we found magnetosome chain fragments composed of 3 to 6 magnetosomes dispersed throughout the cell (Fig. 5b (stars)) which moreover also mostly appear perpendicular to the longitudinal axis of the cell, in agreement with the position of the MamK filament fragments imaged by STED (Fig. 5a, Supplementary Fig. S8). Moreover, the magnetosome fragments are parallel and possess gaps in between (cross). Hence, there is no consistent chain anymore. It has to be noted that the addition of the fluorescent protein TagRFP657 on MamK might influence the elasticity and fragility of the filament. Experiments as those performed by synchrotron-based X-ray diffraction^20^ might help understanding if the mechanics of the MamJ/MamK pair is changed or not.

## Conclusion

In summary, we developed a new FRET pair that allows concomitant STED imaging of one of its constituents in living cells. In general, STED microscopy in living cells is critical due to photodamage and/or phototoxicity^9^. Depending on which type of FPs or organic dyes is used, this might result in lower phototoxicity. However, this strongly depends on the expression level, the extinction coefficient, quantum yield and the used setup fitting to the optical properties of the fluorophores. The used fluorescent proteins in our study, TagRRFP657 as well as phiYFP show low phototoxicity. Thus they are suitable for live cell experiments. Additionally, we have chosen TagRFP657, because as a far-red fluorescent protein it combines the advantage of being less phototoxic and more photostable^32^. The STED images of the control experiment before and after a 20 min regeneration period showed the same structure and a non-significant change in *I*_F,_ indicating an intact cell behavior and minimal photobleaching. FLIM-FRET measurements after magnetic treatment were performed directly or after STED imaging. In the second case, after STED imaging, the donor decay time did not increase. This would happen, when the acceptor is photobleached by STED laser. Thus, no photodamage is recorded on the ongoing MamK (additional discussion in SI).

We have studied earlier the mechanics of the MamJ/MamK pair using synchrotron-based X-ray diffraction^20^. Here, we focused on the development of a technique based on the correlation of two analytical techniques to ultimately unravel the mechanics of the magnetosome chains.

We have studied earlier the mechanics of the MamJ/MamK pair using synchrotron-based X-ray diffraction^20^. Here, we focused on both aspects, the mechanics of the magnetosome chains and the correlated use of the two analytical techniques.

If the combination of the techniques was recently shown, our study demonstrates the feasibility in complex biological systems. Moreover, the combination of techniques will be of high interest in further fields of research: FRET exemplarily found applications in the fields of neuroscience to study membrane fusion proteins and their interaction^33^. This example has also been studied by STED imaging^34^. However, the combination of the techniques using fluorescent proteins is yet to come. An alternative field of application is magnetogenetics, which aims at the non-invasive control and activation of neurons, e.g. by using biofunctionalized magnetic nanoparticles^35,36^. With our approach, the strength of the interaction between the particles and the membrane could be modulated by an external magnetic field and correlative observed *via* FLIM-FRET. Thereby, a magnetic field can be used to exert either a torque on or heat the membrane (*via* magnetosome control) and finally gate neural activity^36^, while changes in shape and structure that are thought to play for synaptic function^37^ could be visualized by STED microscopy. Therefore, given superresolution microscopes spread and genetic techniques develop, we anticipate the combination of FLIM-FRET and STED will provide an accessible and powerful link between mechano- and cellular biology.

## Experimental Methods

### Cultivation

Strains of *Magnetospirillum gryphiswaldense* MSR-1 (MamJ-phiYFP, TagRFP657-MamK, MamJ-phiYFP + TagRFP657-MamK) were grown under aerobic conditions in 15 mL falcon tubes containing 7 mL of modified flask medium^38^. The medium was supplemented with 50 µg/mL kanamycin. 7 mL medium was inoculated with 5 mL pre-culture. The bacteria were grown for 48 h at room temperature, harvested and suspended in a Britton–Robinson (BR) buffer. In order to determine the growth and the average magnetic orientation of the bacteria (C_mag_^39^), a spectrophotometer (UV-1201V, Shimadzu, Japan) at a wavelength of 565 nm (OD_565_) was used (Supplementary Information, Supplementary Tables S5–S7).

### Sample preparation, measurement conditions and electron microscopy

Bacteria were harvested and resuspended in a BR buffer solution. A drop of the bacterial solution (approx. 5 µL) was placed on a coverslip between two magnets (for alignment of the bacteria) and covered by a buffer solution containing 1% of low-melt agarose (#6351, Carl Roth GmbH, Karlsruhe, Germany). An aluminum plate was built on top of the inverted microscope stage using pillars, in order to prevent any physical contact to the microscope. The sample was placed on the stage and centered to a permanent magnet above it, carried by the aluminum plate. This allowed free rotation around the sample without touching the microscope stage and losing the focus.

For chemical fixation of bacteria, 4% paraformaldehyde (PFA) solution was dropped on the bacteria embedded in the agarose matrix with a perpendicular alignment to the direction of the bacteria of the external magnetic field. For imaging using transmission electron microscopy (TEM) the sample was washed after 1.5 h with Milli-Q water and slightly heated up to 30 °C to release the cells from the agarose matrix. A drop of the bacterial solution was then deposited on a copper grid. Transmission electron micrographs were acquired with a Zeiss EM Omega 912 (Carl Zeiss, Oberkochen, Germany) at an acceleration voltage of 120 kV.

### FLIM-FRET measurements

Fluorescence lifetime imaging microscopy (FLIM) was carried out by using the MicroTime 200 time-resolved confocal fluorescence microscope (PicoQuant, Berlin, Germany). The setup consisted of an inverted microscope (Olympus IX 71, Hamburg, Germany) mounted with an oil immersion objective (Nikon Plan Apo Lambda 100×, NA 1.45). The donor protein phiYFP was excited at λ = 475 nm using a *ps*-pulsed supercontinuum source (SC450-2, Fianium Ltd., Southampton, UK), driven and triggered by an external diode laser driver (PDL 828, PicoQuant) and operating at a repetition rate of *f*_rep_ = 20 MHz with ~30 ps pulse width. The average laser power was adjusted to 3.4 µW at the objective’s back aperture. The excitation light was guided to the sample by a custom-built 3 mm multiband-dichroic mirror zt405/473/532/640/750-1100rpc (AHF Analysentechnik, Tübingen, Germany). After passing a 50 µm pinhole, a long-pass filter LP500 and a short-pass filter SP550 (Supplementary Fig. S9), the fluorescence was collected by a single-photon avalanche diode (SPAD, SPCM-CD-2801, Perkin Elmer, Waltham, USA). Image acquisition occurred by raster-scanning of the objective using a *xyz*-piezo-positioner (Physik Instrumente, Karlsruhe, Germany).

Time-resolved fluorescence recordings were performed in the time-correlated single-photon counting (TCSPC) mode using a PicoHarp 300 (PicoQuant) operating with a time resolution of 8 ps. Data acquisition and analysis were performed by using the software SymPhoTime 64 (PicoQuant, *vers*. 2.1). Fluorescence decay curves were calculated from recorded FLIM images by summing up all photons in a selected region of interest. For the analysis, we seek for reaching a peak maximum of 1000. Decay curves were analyzed by deconvolution fits using the instrument response function (IRF), which was measured daily with mean FWHM of 460 ± 19 ps (*N* = 10). In control experiments, the fluorescence decay time of the donor in absence of the acceptor (τ_D_) was determined by applying a *single-exponential decay* function. For the FRET samples, a bi-exponential fit function was assumed. Here, the first time component was fixed according to τ_D_ previously obtained from the control experiments and reflecting the non-interacting donor fraction and the second time component τ_DA_ was freely fitted reflecting the donor fraction interacting with the acceptor. The quality of the fit was assessed by randomly distributed residuals and by low ${\chi }_{R}^{2}$ values. Exemplary fitting curves and corresponding residuals are shown in the supplementary (Supplementary Figs. S11–S14. From the interacting fraction, the Förster resonance energy transfer (FRET) efficiency *E* was calculated according to^40^:

1 $$E=1-(\frac{{\tau }_{DA}}{{\tau }_{D}})$$

The spectral overlap integral *J(λ*) in M^−1^ cm^−1^ nm^4^ is given by:

2 $$J(\lambda )={\int }_{o}^{\infty }\,{F}_{D}(\lambda )\,{\varepsilon }_{A}(\lambda )\,{\lambda }^{4}\,d\lambda $$

where *F*_D_(*λ*) is the normalized emission spectrum of the donor, *ε*_A_(*λ*) is the molar absorptivity of the acceptor and the wavelength *λ* (Supplementary Fig. S10). From this, the Förster distance *R*_0_ is given by:

3 $${R}_{0}=\sqrt[6]{\frac{9\,(\mathrm{ln}\,10)\,{\kappa }^{2}{\varphi }_{D}}{128\,{\pi }^{5}{N}_{A}\,{n}^{4}}\,J(\lambda )}\,$$

where *N*_A_ is the Avogadro constant 6.022 × 10^23^ mol^−1^, *κ*^2^ is the orientation factor between the FRET pair and is assumed to be 2/3 for random oriented fluorophores, *n* is the refractive index with *n* = 1.36 for MSR-1 cells^41^, *Φ*_D_ is the fluorescence quantum yield of the donor phiYFP with *Φ*_D_ = 0.60^42^. Finally, the donor-acceptor distance *r* can be then calculated according to:

4 $$r=\,{R}_{0}\sqrt[6]{\frac{1-E}{E}}$$

### STED microscopy

Diffraction-unlimited imaging was implemented in the same microscope by using stimulated emission depletion (STED). The acceptor protein TagRFP657 was excited at λ = 635 nm using a ~90 ps-pulsed laser diode (LDH-P-635, PicoQuant). For STED depletion at λ = 765 nm, a ~80 ps-pulsed laser diode was applied (LDH-P-FA-765, PicoQuant). The lasers were controlled by a diode laser driver (PDL 828, PicoQuant) and the repetition rate of both lasers was reduced to *f*_rep_ = 2.5 MHz. The STED laser was guided *via* a PM-fiber to a spatial light modulator (Pluto-NIR-015-C, Holoeye Photonics, Berlin, Germany) and by application of specific phase masks on the modulator, a well-defined STED-donut light distribution was created^43^. The phase-modulated STED light passed then a Glan-Thompson polarizer and was exactly overlapped with the excitation light by the inverted dichroic 725dcspxr (AHF Analysentechnik). After passing an achromatic λ/4-plate, changing the polarization state to circular, the 635 nm and 765 nm laser light was guided to the objective *via* the multiband-dichroic mirror as used for FLIM-FRET. All other parts of the microscopic setup are identical to that of FLIM-FRET, except for the emission filter BP690/70 and the SPAD used (SPCM-AQR-13, PerkinElmer, Waltham, USA) (Supplementary Fig. S8). The STED laser power was adjusted to *P*_av_ = 2.5 mW at the objective’s back aperture and the time delay between excitation and depletion laser was set to approx. 300 ps. Image acquisition occurred in the TCSPC mode, thus STED images could be generated by time-gating and ignoring early photons from the lasers^44^. Signal-to-noise ratio of STED images was additionally improved by deconvolution based on a Richard-Lucy algorithm using a freely available ImageJ plugin, Deconvolution Lab2^45^. For better visibility, 3D surface intensity plots were calculated from STED images by using Gaussian filtering (σ = 2) in Matlab (vers. 9.0, The MathWorks Inc., Massachusetts, USA).

### Data analyses

Data were tested for normal distribution using D’Agostino and Pearson omnibus normality test (P < 0.05). In a case of non-normality, a Kruskal–Wallis test followed by Dunn’s multiple comparisons tests were performed for comparing more than two data sets. In a case of normal distribution, two data sets were statistically analyzed by a parametric paired *t*-test. Differences were considered statistically significant, if *P* < 0.05. Statistical analyses were performed using Origin (vers. 9.1, OriginLab, Northampton, USA).

## Supplementary information

Supplementary information
