# Supplementary material for: The in vivo mechanics of the magnetotactic backbone as revealed by correlative FLIM-FRET and STED microscopy
Source: Sci Rep. 2019 Dec 23;9:19615. doi: 10.1038/s41598-019-55804-5 (PMC6928011; doi:10.1038/s41598-019-55804-5)
Supplement: Supplementary file 1 — Supplementary information [file 41598_2019_55804_MOESM1_ESM.pdf]

# The *in vivo* mechanics of the magnetotactic backbone as revealed by correlative FLIM-FRET and STED microscopy

*Erika Günther*[1], *André Klauß*[2,4], *Mauricio Toro-Nahuelpan*[3,5], *Dirk Schüler*[3], *Carsten Hille*[2,6], *Damien Faivre*[1,7,*]

[1]Department of Biomaterials, Max Planck Institute of Colloids and Interfaces, Science Park Golm, 14424 Potsdam, Germany

[2]Physical Chemistry, Institute of Chemistry, University of Potsdam, 14476 Potsdam, Germany

[3]Department of Microbiology, University Bayreuth, 95447 Bayreuth, Germany

[4]Present address: *HOLOEYE* Photonics AG, 12489 Berlin, Germany

[5]Present address: European Molecular Biology Laboratory, 69117 Heidelberg, Germany

[6]Present address: Technical University of Applied Sciences Wildau, 15745 Wildau, Germany

[7]Aix Marseille University, CEA, CNRS, BIAM, 13108 Saint Paul-Lez-Durance, France

[*] **e-mail: damien.faivre@mpikg.mpg.de.**

## Supplementary information

### Control experiments

We acquired two consecutive FLIM-FRET and STED images of the same cells. First, we verified changes in distance between MamJ-phiYFP and TagRFP657-MamK using FLIM-FRET and in the structure of the filament of TagRFP657-MamK by STED microscopy. The bacteria are embedded in an agarose matrix to keep them alive, but also to prevent them from being able to freely move and in particular rotate. We performed FLIM-FRET and STED microscopy on a bacterium and after 20 min regeneration period, the same procedure was repeated on the same cell. Supplementary Figure 2a shows the STED image of three cells (TagRFP675-MamK filaments). Supplementary Figure 2b shows the identical cells after 20 min regeneration period. Both images display consistent and straight filaments.

In addition, using equation (1), we calculated the FRET efficiency at the beginning of the experiment $E = 78 \pm 5\%$ (n = 14 cells, N = 10 images, 2 independent experiments / imaging of different cultures and at different days). After 20 min regeneration period, the FRET efficiency is $E = 82 \pm 3\%$ (n = 13 cells, N = 10 images, 2 independent experiments). The calculated distance between the donor phiYFP and acceptor TagRFP657 using equation (4) is $r = 3.7 \pm 0.2$ nm (n = 14, N = 10) and $r = 3.4 \pm 0.2$ nm (n = 13, N = 10) for the repeated measurement after 20 min. The FLIM-FRET results demonstrate that the distance of MamJ-phiYFP and TagRFP657-MamK change slightly during STED imaging and the 20 min regeneration period in a range of $\Delta r = 0.3$ nm, which is statistically significant ($P < 0.01$), but physiologically not relevant. Additionally, the STED images of the TagRFP657-MamK filament are similar (see also Supplementary Figure S2).

**Measured fluorescence decay times, calculated FRET efficiencies and donor-acceptor distances**

We measured the fluorescence decay time (τ) of the donor phiYFP in presence and absence of the acceptor TagRFP657 by time-correlated single-photon counting (TCSPC). Decay curves from MamJ-phiYFP strains were successfully fitted ($\chi_R^2$ = 1.28) to a single-exponential decay function with a resulting decay time $\tau_D$ = 2.95 ns. Decay curves of MamJ-phiYFP + TagRFP657-MamK strains show a faster decay compared to the MamJ-phiYFP strains. We quantified this decay behavior by a bi-exponential decay function ($\chi_R^2$ = 1.10), determining the interacting and non-interacting donor populations, resulting in a second decay time $\tau_{DA}$ = 0.56 ns (with a fixed decay time $\tau_D$ = 2.94 ns). Supplementary Tab. 1 summarizes the calculated fluorescence decay times of the donor phiYFP of different MSR-1 strains and statistical analysis. No significant change is observed between the fluorescence decay times $\tau_D$ of MamJ-phiYFP and phiYFP + TagRFP657-MamK constructs. Thus, the presence of donor and acceptor fluorescent proteins alone is not sufficient for FRET events. However, in the presence of donor and acceptor fluorescent proteins fused to MamJ and MamK, the fluorescence decay times decrease significantly, but independent of the mechanical treatment (see also Supplementary Tab. 1).

**Energy transfer between MamJ-phiYFP and TagRFP657-MamK**

MamJ-phiYFP + TagRFP657-MamK was excited up to 4 times longer than MamJ-phiYFP or phiYFP + TagRFP657 to get sufficient counts for analyzing, indicating the successful energy transfer from the donor (phiYFP) in presence of the acceptor (TagRFP657). On the other hand, direct acceptor excitation with the same power at 635 nm did not result in increased acceptor

intensity in MamJ-phiYFP + TagRFP657-MamK due to the low quantum yield (0.1) of TagRFP657.

**Fitting procedure**

When applying time-resolved donor fluorescence measurements for studying FRET interactions, the measurements provide access to non-interacting and interacting fractions of the donor molecules – a benefit compared to intensity-based FRET recordings. Thus, the standard procedure is then divided into two steps[1]. First, the donor fluorescence decay time is recorded alone ($\tau_D$) and subsequently in the presence of the acceptor ($\tau_D$). Figure 1a shows the mono-exponential decay behavior of the donor alone (yielding $\tau_D$ with $\chi_R^2$-values of 1.28, Supplementary Fig. S11). In the presence of donor and acceptor without interaction (control), the donor decay is not changed (still mono-exponential decay behavior), indicating the absence of influence of the fluorescent acceptor on the donor (see Supplementary Tab. S3, second row, added Supplementary Fig. S14).

However, in the case of a possible interaction between donor and acceptor, we clearly saw a multi-exponential donor fluorescence decay behavior (see Figure 1, Supplementary Fig. S14). Since we only detected donor molecules, the simplest way was to assume non-interacting and interacting donor molecules. Since non-interacting donor exhibited a mono-exponential decay time of 2.95 ns (which did not change under the presence of non-interaction acceptor), we fixed this value (only the time component, but not the fractional contribution). The second time component then corresponded to the interacting donor with reduced decay time originating from the energy transfer to the acceptor. Thus, bi-exponential decay fitting with only one fixed time component resulted then in the second time component (indicating the FRET efficiency) and in the fractional contribution (indicating the amount of interacting donor relative to all available donor molecules).

The determined IRF value (460 ps) falls within common range for this type of experimental setup (and is mainly determined by the SPAD-detector timing[2]. Theoretically, temporal resolutions down to 1/10 of the IRF can be reached, but in practice values of ½ of the IRF are realistic and distinguishable[3]. Thus, the time components we obtained could be analyzed with sufficient accuracy. In addition, the residuals and $\chi_R^2$-values indicated a reliable fitting procedure.

**Autofluorescence contribution**

A significant fluorescence signal under the chosen experimental conditions could not be observed in cells lacking the donor or acceptor (Supplementary Fig. S1c, e). Thus, autofluorescence was neglected.

**STED microscopy on MamK filaments**

After one STED round, we observed a certain structure, but the image had an insufficient quality (in term of counts). Accordingly, we waited 20 min in order to compare two consecutive STED images/ filaments. In the interval, the structure is likely reformed since, after 20 min of regeneration time, we observed again a fluorescence signal. We hypothesized the new fluorescent filament originated from treadmilling. We therefore concluded that the cells were still alive and that no significant phototoxicity took place.

**High FRET efficiencies, collection of photons**

Supplementary Tab. S3 shows that approximately 73 % to 81 % are interacting donor molecules. In general, not all donor molecules are able to interact with the acceptor[1]. However, upon the presence of a dynamic and intact MamK filament, or a stiff and static filament, the MamJ turnover remains constant[4]. Consequently, the dynamic behavior of MamJ is independent of MamK and is

proposed to only transiently interact with MamK. The half time of recovery of MamJ fusion proteins after photobleaching is approximately 10 s [4] and thus indicates a transient MamJ-MamK interaction < 20 s [4]. All photons were collected in an image obtained within approximately 50 s for the fluorescence decay fitting in the FLIM-FRET analysis, since faster point and line scans resulted in insufficient photon statistics. Consequently, short time interactions are averaged out for numerical FRET analysis. However, during the scanning process, interacting and non-interacting donor populations were visualized. On the one hand, free MamJ and MamK in the cytoplasm could result in this occurrence. On the other hand, under the assumption that the MamJ-MamK interactions are < 20 s, an inhomogeneous decay time distribution in a FLIM image recorded within 50 s could be considered. Hence, this distribution would reflect the non-synchronized and transient interaction in a bacterium, as observed in the presented results.

**Normalization of TagRFP657 fluorescence intensities**

The images were normalized to maximum count value for comparison. Indeed, the counts refer as arbitrary unit to the recorded fluorescence intensity per pixel (Figure 4c: 0 - 84 counts per pixel, normalized to 84 counts = 1). Nevertheless, the changed intensities did not totally refer to bleaching, but also to slight changes in position and / or focus, to changed protein expression and/ or distribution, and to variations due to the microscope setup, since we measured in sub-diffraction space. Still, the counts were high enough to produce a meaningful image.

**Plasmid construction**

Plasmids were constructed by amplifying the DNA fragments of interest with the Phusion High Fidelity DNA Polymerase (Thermo Scientific). Plasmid description and oligonucleotides are listed

in Supplementary Tab. 5 to 7, respectively (see below). Plasmids were introduced into *M. gryphiswaldense* by conjugation.

To generate pMT092, the *phiYFP* gene (Evrogen) was amplified with the oligonucleotides oMTN292 and oMTN293. Subsequently, the *phiYFP* fragment was *Hind*III-*Bam*HI digested and ligated into the identically digested pMT082 vector.

To construct pMT093, the *TagRFP657* (Addgene) gene was amplified (oMTN294-295) and further cloned into the vector pMT080 under the control of the *mamAB* operon promoter ($P_{mamAB}$) using the restriction sites *Nde*I-*Eco*RI.

Plasmid pMT094 was created by amplifying the *mamK* gene (oMTN045-296) and subsequently cloned into pMT093 by using the *Hind*III-*Bam*HI restriction sites.

For creation of pMT095, the *TagRFP657-mamK* fragment was amplified (oMTN297-298) and *Bam*HI-*Xho*I digested to be cloned into identically digested pMT092 vector.

For construction of pMT107, the *phiYFP* + *TagRFP657-mamK* fragment was amplified (oMTN353-355) and cloned into pMT094 using the *Nde*I-*Bam*HI restriction sites.

Finally, to construct pMT108, the vector pMT107 was *Bam*HI-*Xho*I digested. Subsequently, blunt ends were generated and further re-ligated (in addition see Supplementary Tab. 5 - 7).

The fusions MamJ-phiYFP, TagRFP657-MamK, and MamJ-phiYFP + TagRFP657-MamK rescued the phenotypes of the $\Delta$*mamJ*, $\Delta$*mamK, and* $\Delta$*mamJK* strains, respectively, which exhibited wild type-like magnetosomes chains (data not shown). This means that the fusions are functional.

# Supplementary figures

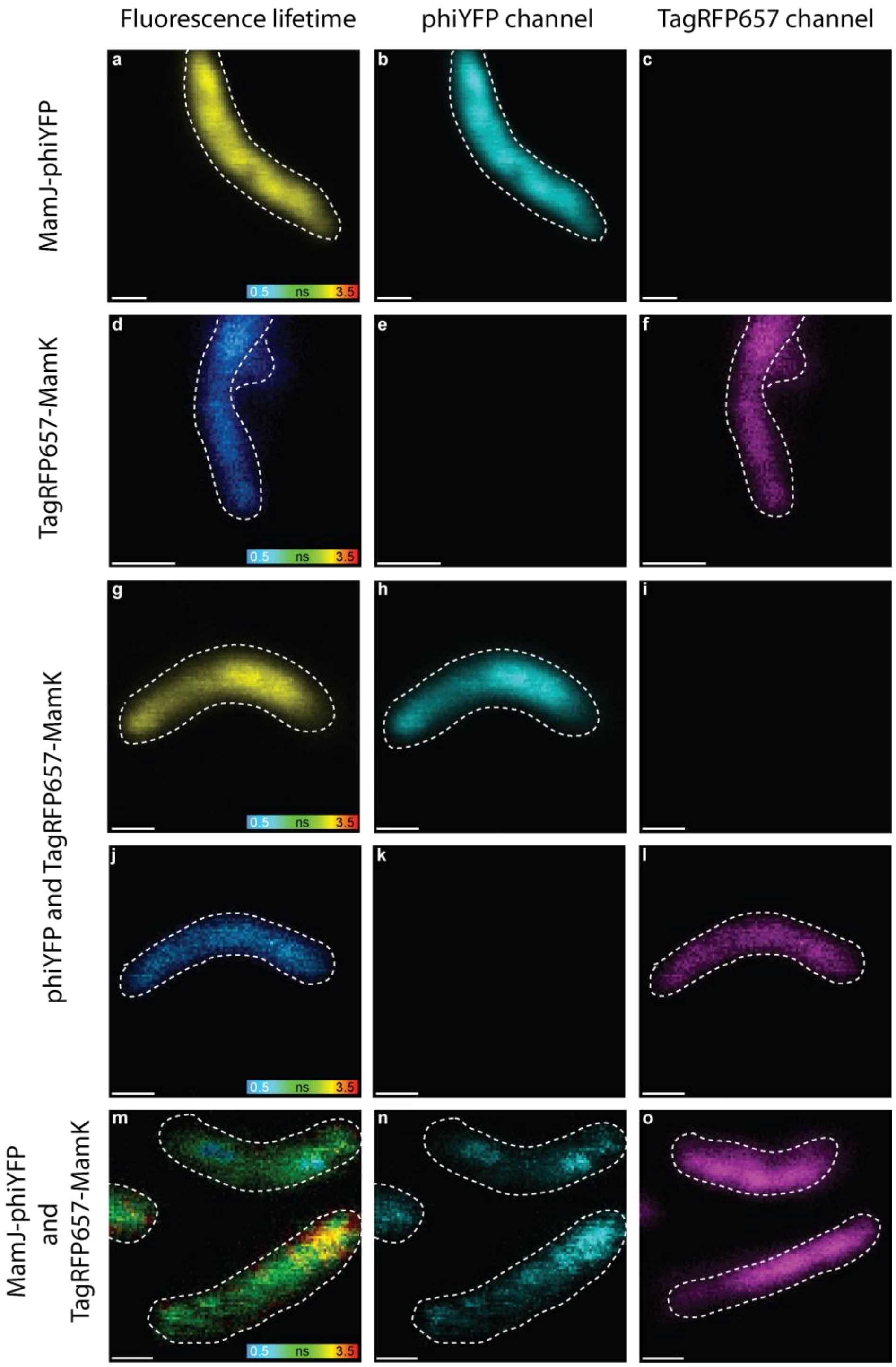


Supplementary Figure S1**: Fluorescence lifetime and fluorescence intensity images of MamJ-phiYFP, TagRFP657-MamK and MamJ-phiYFP + TagRFP657-MamK in the phiYFP**

**channel and TagRFP657 channel.** The first row shows MSR-1 cells with only MamJ-phiYFP (a-c) with the fluorescence lifetime image of the phiYFP-channel colored in yellow (a) indicating a long donor decay time. The fluorescence intensity image is colored in cyan (b). The second row illustrates MSR-1 cells with only TagRFP657-MamK (d-f) with the fluorescence lifetime image of the TagRFP657-channel colored in blue (d) indicating a short acceptor decay time. The fluorescence intensity image is colored in purple (f). The third and fourth row shows MSR-1 cells with TagRFP657-MamK and phiYFP (g-l) with the fluorescence lifetime image of the phiYFP-channel colored in yellow (g) indicating a long donor decay time and with the fluorescence lifetime image of the TagRFP657-channel colored in blue (j) indicating a short acceptor decay time. The fluorescence intensity of phiYFP is colored in cyan (h) and the fluorescence intensity of TagRFP657 is colored in purple (l). The fifth row shows MSR-1 cells with MamJ-phiYFP and TagRFP657-MamK (m-o) with the fluorescence lifetime image of the phiYFP-channel (m), in which green colors indicate the reduced donor decay time due to energy transfer. The fluorescence intensity of phiYFP is colored in cyan (n) and the fluorescence intensity of TagRFP657 is colored in purple (o).

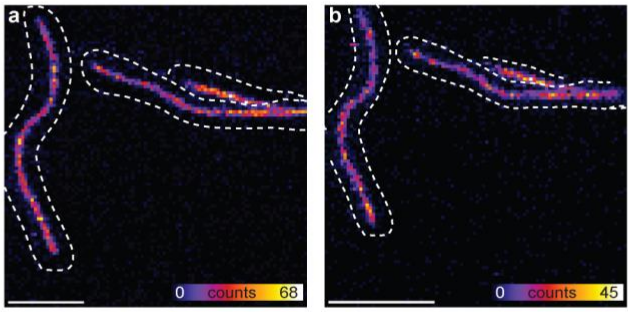


Supplementary Figure S2: **STED images of MSR-1 cells expressing MamJ-phiYFP + TagRFP657-MamK.** The fluorescence intensity of TagRFP657 was recorded, indicating the filament TagRFP657-MamK. First image scan (a) second image scan after 20 min regeneration period (b). Scale bar: 1 µm.

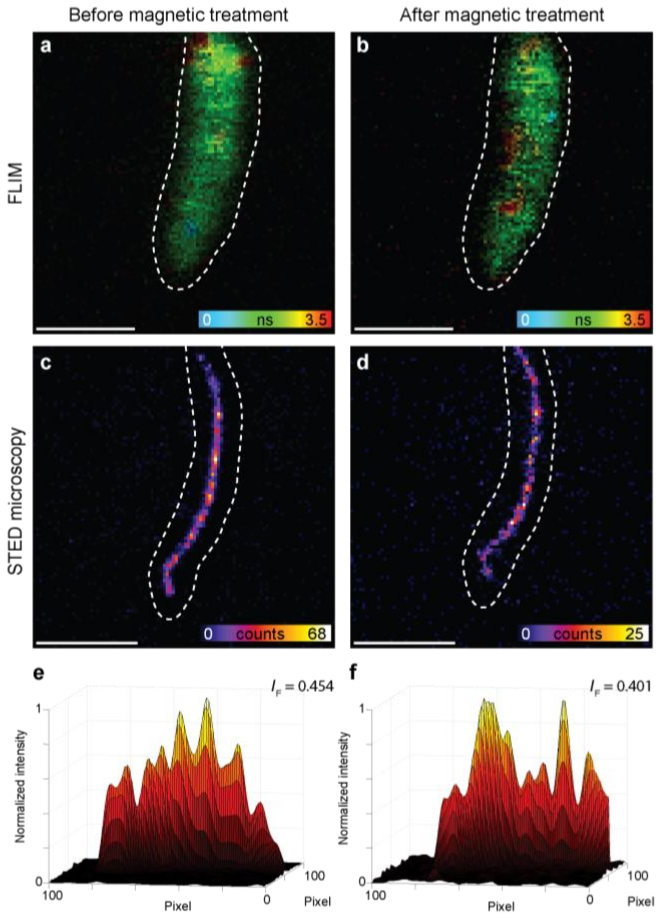


Supplementary Figure S3: ***In vivo* FLIM-FRET and STED experiments on MSR-1 cells before and after magnetic field rotation.** FLIM images (a, b) and STED images (c, d) of MSR-1 cells with MamJ-phiYFP + TagRFP657-MamK before (a, c) and after (b, d) applying a magnetic field. (e, f) Corresponding 3D surface intensity plots of the STED images. For a better illustration, we normalized the intensity and used a Gaussian filter.

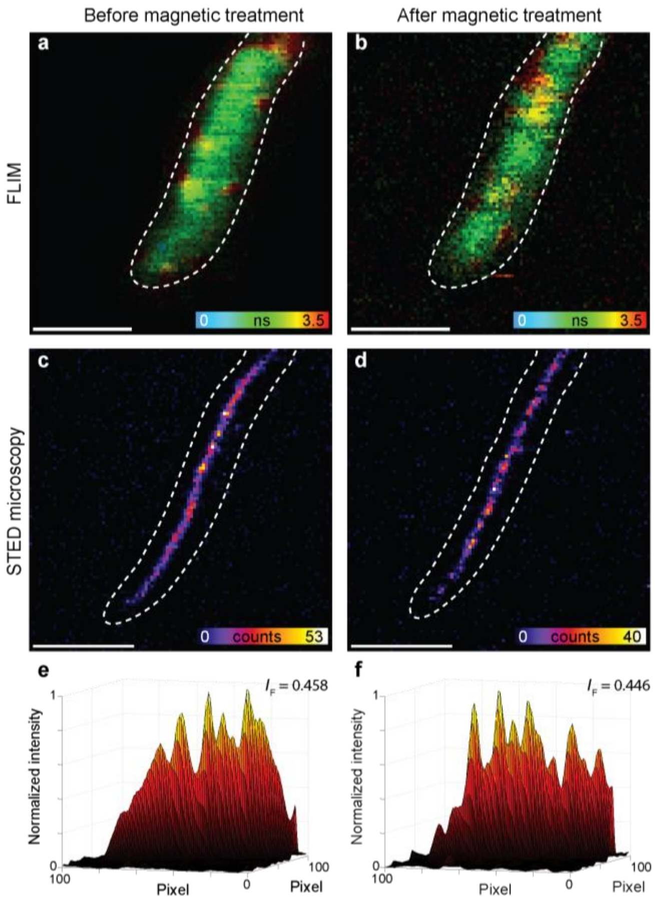


Supplementary Figure S4: ***In vivo* FLIM-FRET and STED experiments on MSR-1 cells before and after magnetic field rotation.** FLIM images (a, b) and STED images (c, d) of MSR-1 cells with MamJ-phiYFP + TagRFP657-MamK before (a, c) and after (b, d) applying a magnetic field. (e, f) Corresponding 3D surface intensity plots of the STED images. For a better illustration, we normalized the intensity and used a Gaussian filter.

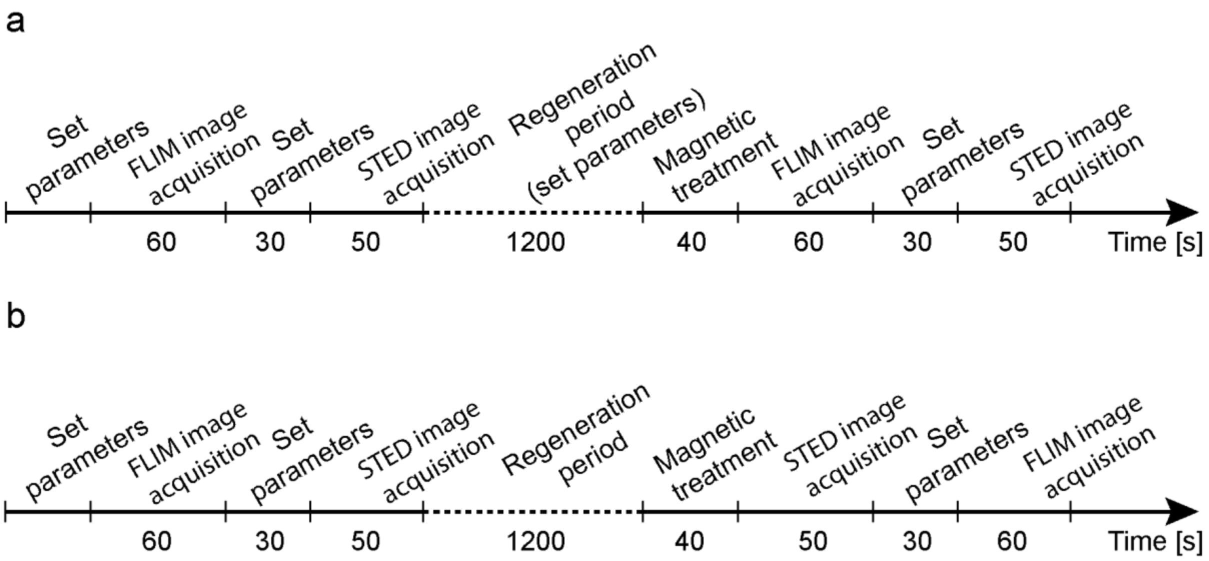


Supplementary Figure S5: **Timeline of the image acquisition.** (a) The FLIM image recording time is approx. 60 s followed by approx. 50 s for the STED imaging. In between is a short break of approx. 30 s to change the setting for the next measurement. This procedure is repeated after a 20 min regeneration period and the magnetic treatment. (b) Immediately after the regeneration period and the magnetic treatment, the STED image was recorded followed by the FLIM image acquisition.

The FRET efficiency did not change after magnetic treatment. Therefore, we changed the order of the image acquisition to reduce the time for the *de novo* synthesis of MamK.

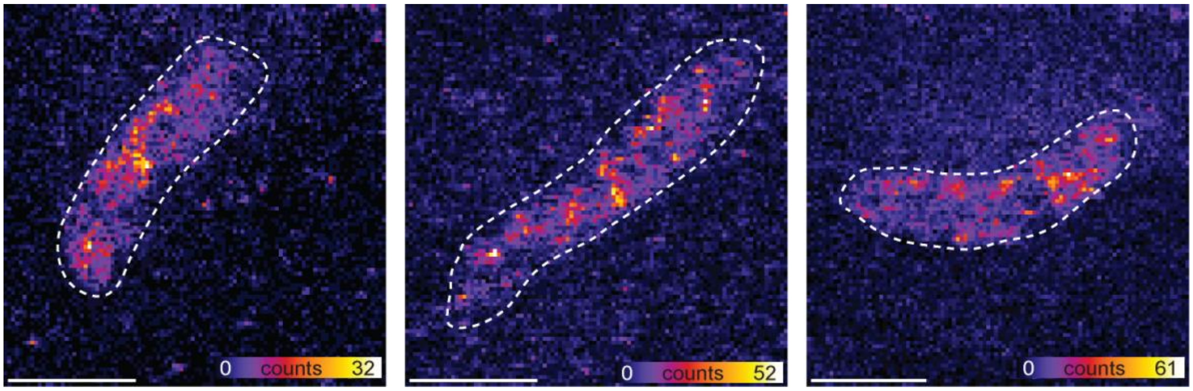


Supplementary Figure S6: **STED images of filament fragments after rotating a magnetic field and chemical fixing of the cells with 4% PFA. Scale bar: 1 μm.**

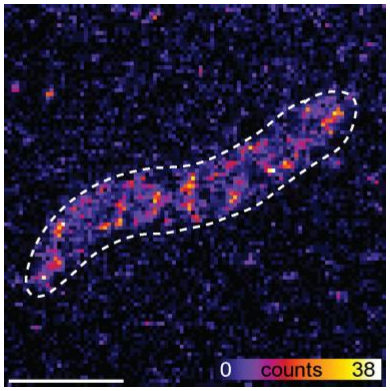


Supplementary Figure S7: **STED images of filament fragments after rotating a magnetic field and chemical fixing of the cells with 4% PFA. Scale bar: 1 µm.**

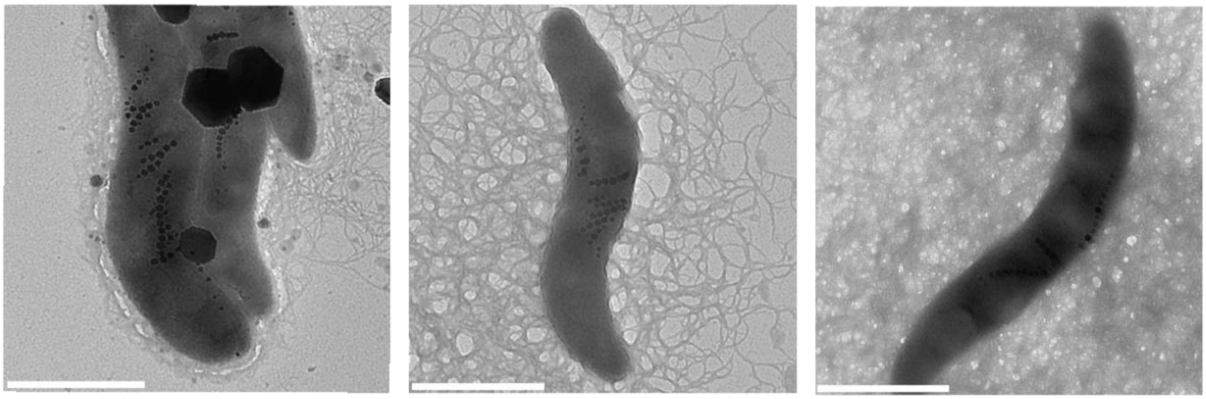

Supplementary Figure S8: **TEM images of chain fragments after rotating a magnetic field and chemical fixing of the cells with 4% PFA. Scale bar: 1 µm.**

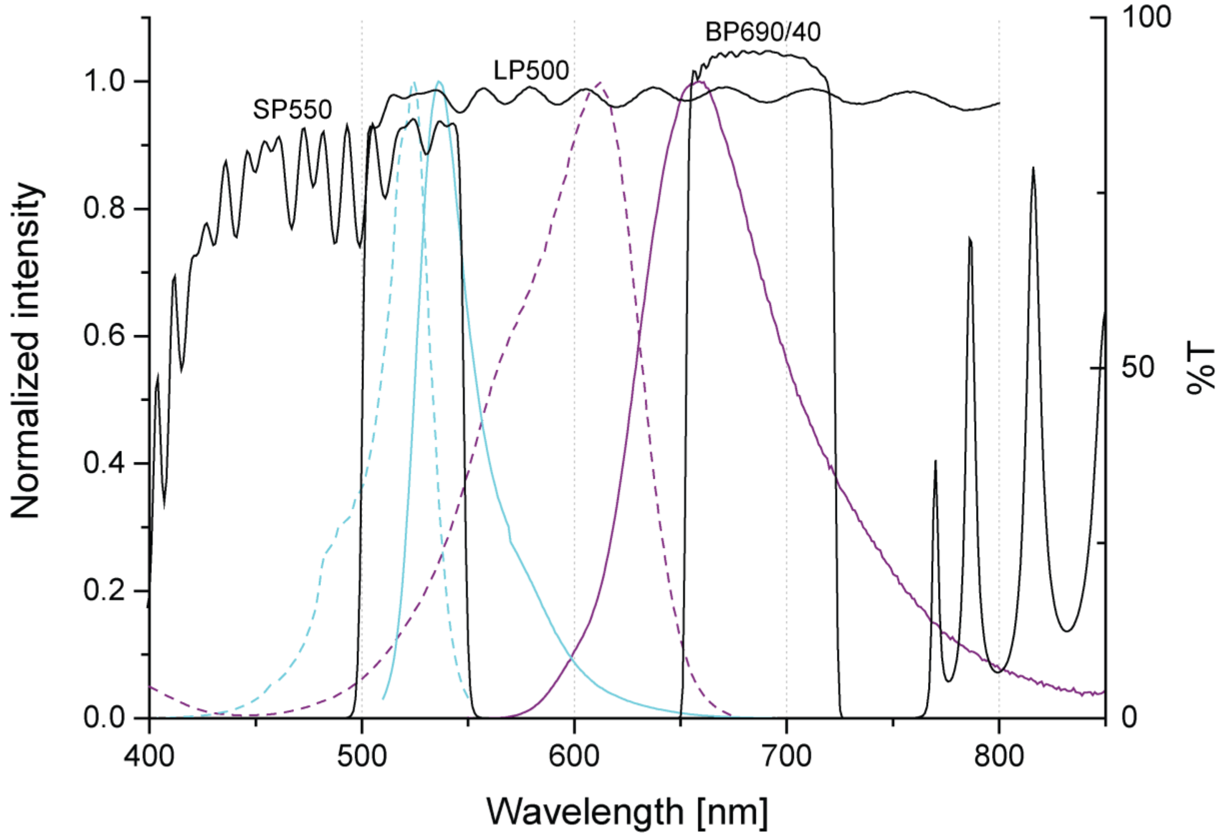


Supplementary Figure S9: **Absorption (dashed lines) and emission (solid lines) of phiYFP (cyan) and TagRFP657 (purple) as well as transmission curves of the chosen filters (black) for FLIM-FRET and STED experiments.**

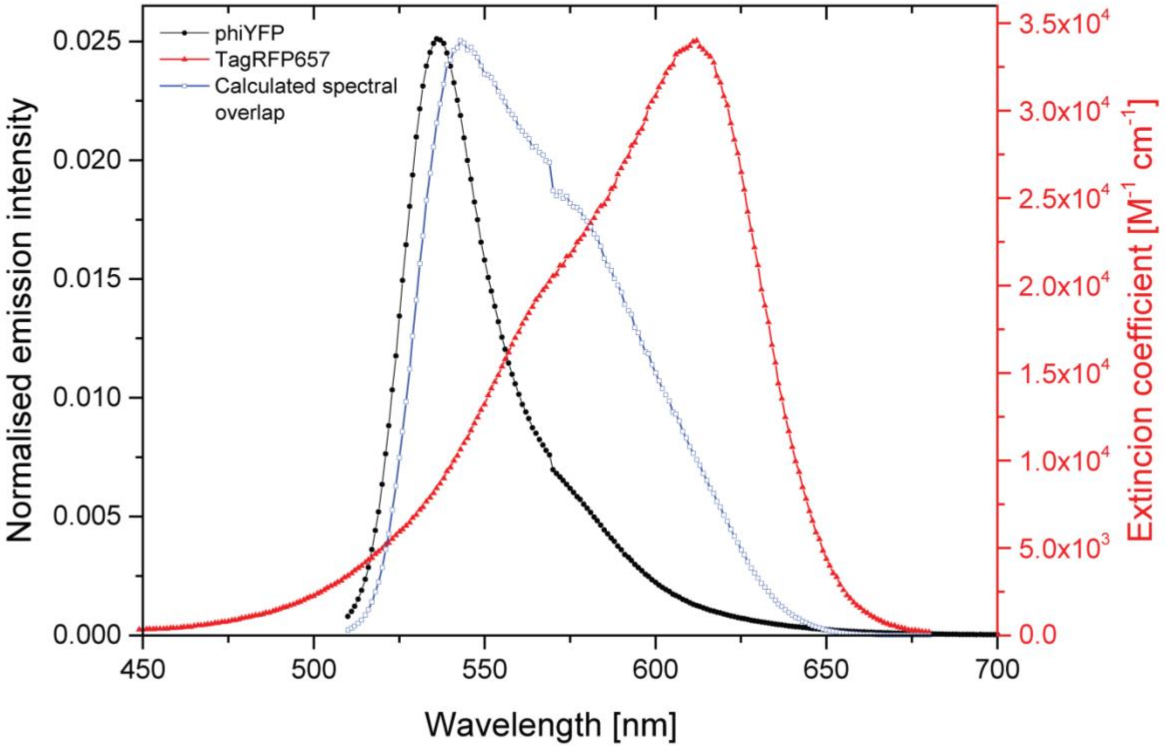


Supplementary Figure S10: **Area-normalized emission spectrum of phiYFP (black), molar absorption spectrum of TagRFP657 (red) and resulting overlap function indicating the spectral overlap integral *J*($\lambda$).**

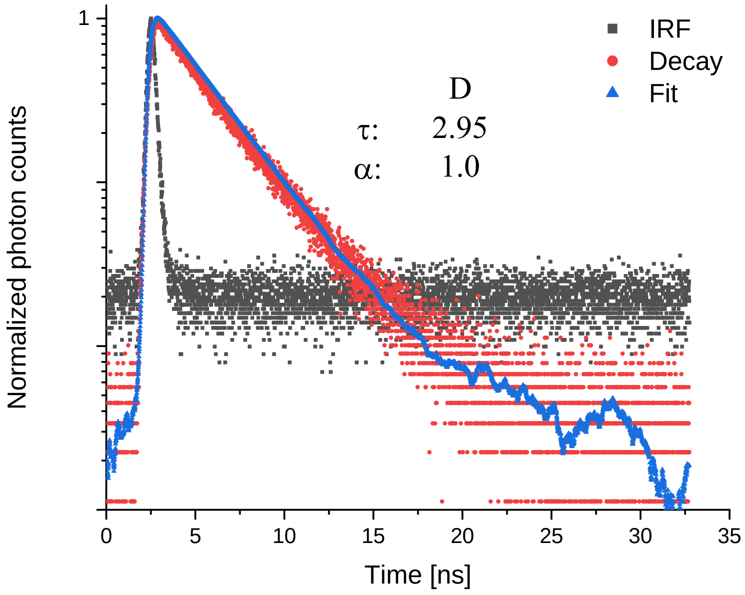


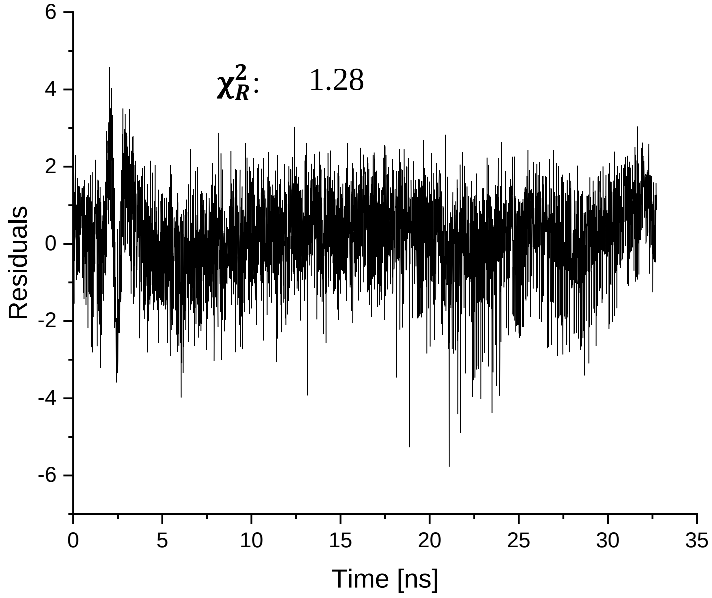


Supplementary Figure S11: ***In vivo* FLIM-FRET analysis in MSR-1 cells with MamJ-phiYFP**. A representative phiYFP (donor) fluorescence decay curve was measured by time-correlated single-photon counting (TCSPC) in MSR-1 expressing MamJ-phiYFP (red data points). For MamJ-phiYFP, the data was fitted to a single-exponential deconvolution fitting model (blue),

yielding the decay time $\tau_D$ = 2.95 ns. The instrument response function (IRF) is shown in grey. The amplitude ($\alpha$) is 1.0. The corresponding $\chi_R^2$ is displayed below (1.28).

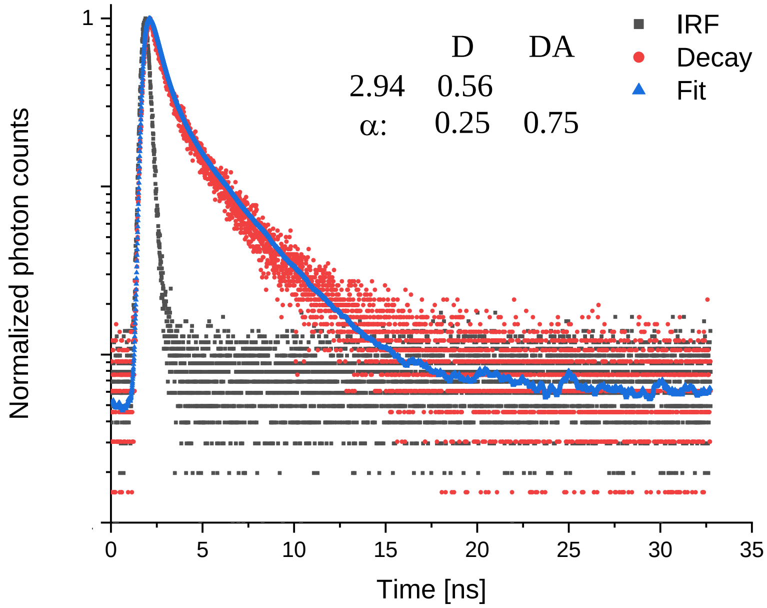


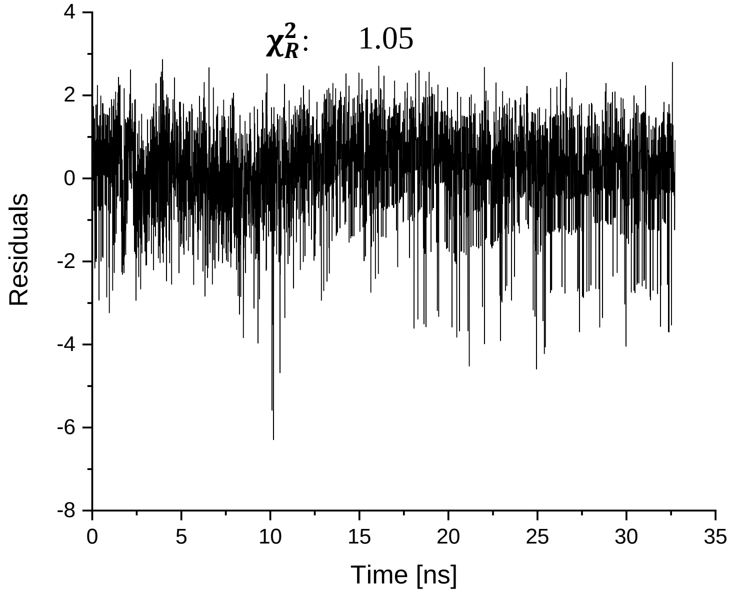


Supplementary Figure S12: ***In vivo* FLIM-FRET analysis in MSR-1 cells with MamJ-phiYFP and TagFRP657-MamK**. A representative fluorescence decay curve was measured by time-correlated single-photon counting (TCSPC) in MSR-1 expressing MamJ-phiYFP and TagRFP657-

MamK (red data points). The data was fitted to a bi-exponential deconvolution fitting model (blue line), yielding the decay time $\tau_D$ = 2.94 ns and an amplitude ($\alpha_D$) of 0.25 along with $\tau_{DA}$ = 0.56 ns and an amplitude ($\alpha_{DA}$) of 0.75. The instrument response function (IRF) is shown in grey. The corresponding $\boldsymbol{\chi_R^2}$ is displayed below (1.05).

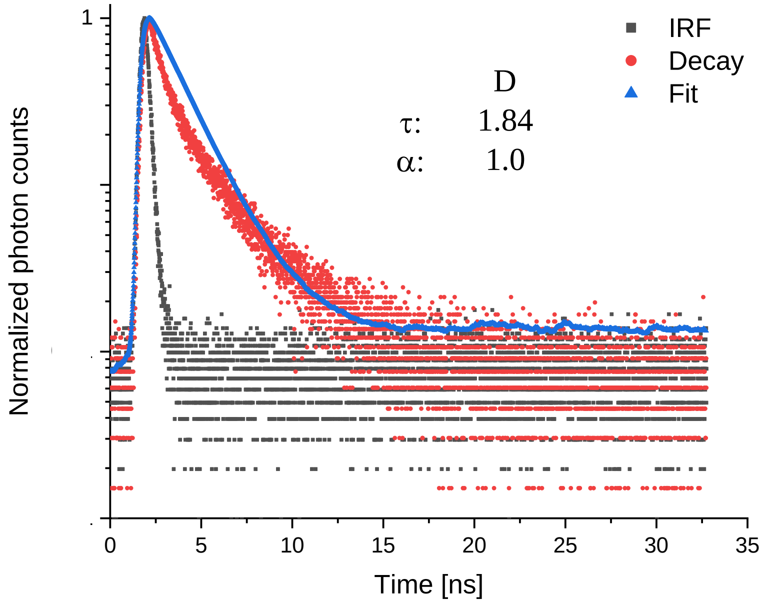


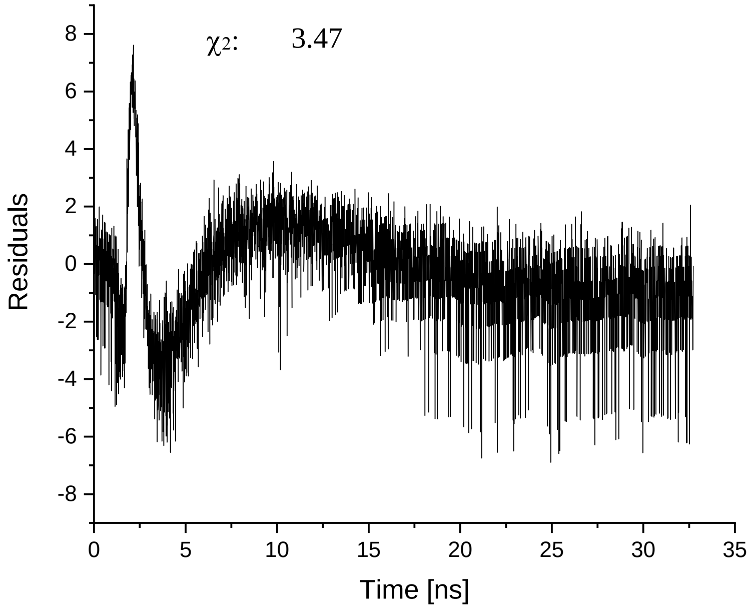


Supplementary Figure S13: ***In vivo* FLIM-FRET analysis in MSR-1 cells with MamJ-phiYFP and TagFRP657-MamK.** A representative fluorescence decay curve measured by time-correlated single-photon counting (TCSPC) in MSR-1 expressing MamJ-phiYFP and TagRFP657-MamK

(red data points). The data was fitted to a single-exponential deconvolution fitting model (blue line), yielding the decay time $\tau_D$ = 1.84 ns and an amplitude ($\alpha_D$) of 1.0. The instrument response function (IRF) is shown in grey. The corresponding $\boldsymbol{\chi_R^2}$ is displayed below (3.47).

The fitting of the decay curve using a bi-exponential deconvolution fitting model resulted in a better $\chi_R^2$. Thus we used the bi-exponential deconvolution fitting model for MSR-1 cells expressing MamJ-phiYFP and TagRFP657-MamK.

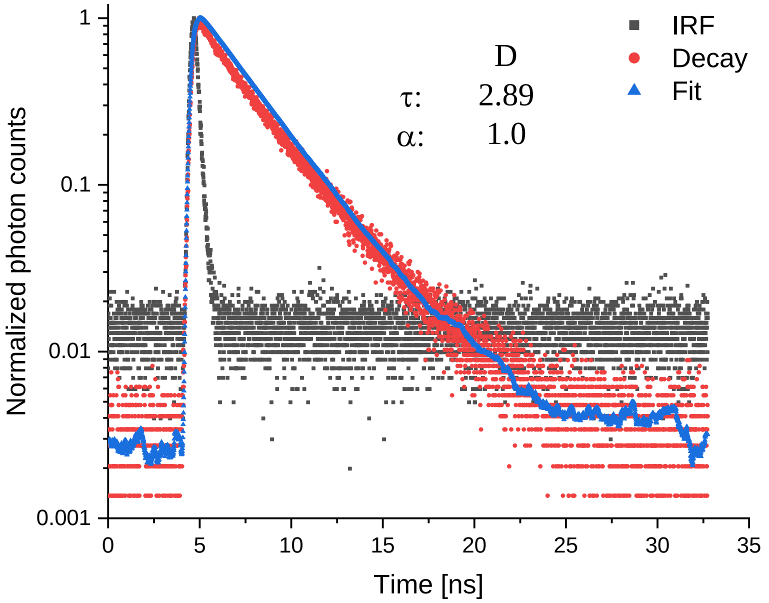


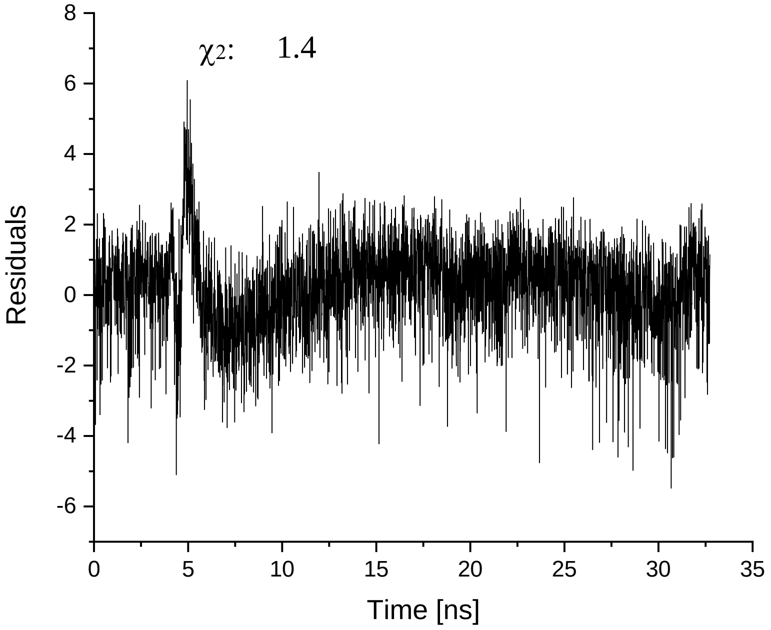


Supplementary Figure S14: ***In vivo* FLIM-FRET analysis in MSR-1 cells with phiYFP and TagFRP657-MamK.** A representative fluorescence decay curves measured by time-correlated single-photon counting (TCSPC) in MSR-1 expressing phiYFP and TagRFP657-MamK (red data

points). The data was fitted to a single-exponential deconvolution fitting model (blue), yielding the decay time $\tau_D$ = 2.89 ns and an amplitude ($\alpha_D$) of 1.0. The instrument response function (IRF) is shown in grey. The corresponding $\boldsymbol{\chi_R^2}$ is displayed below (1.4).

**Supplementary tables**

Supplementary Table S1: **Full-width at half maximum (FHWM) of confocal and STED fluorescence image of TagRFP657-MamK filaments.**

| Bacterium | Confocal [nm] | STED [nm] |
|---|---|---|
| Figure 1d | 264 | 60 |
| Supplementary Figure S4c | 220 | 66 |
| Supplementary Figure S4c | 217 | 63 |
| 4* | 233 | 84 |
| 5* | 200 | 75 |
| 6* | 261 | 54 |
| 7* | 225 | 77 |
| 8* | 202 | 102 |
| 9* | 203 | 73 |
| 10* | 207 | 80 |

*Images are not shown.

The FWHM was determined at the center of the cell. The mean FHWM of the confocal fluorescence images of MamK-TagRFP657 is 223 ± 23 nm and was significantly reduced to 73 ± 24 nm in STED fluorescence images ($P < 2 \cdot 10^{-7}$).

Supplementary Table S2: **Determined area of the MamK filament before and after a 20 minute regeneration period.**

| Filament | $I_F$ before magnetic treatment | $I_F$ after magnetic treatment |
|---|---|---|
| Supplementary Figure S2 | 0.533 | 0.501 |
| Supplementary Figure S2 | 0.369 | 0.366 |
| Supplementary Figure S2 | 0.591 | 0.522 |
| 4* | 0.477 | 0.442 |
| 5* | 0.527 | 0.397 |
| 6* | 0.49 | 0.491 |
| 7* | 0.486 | 0.393 |
| 8* | 0.376 | 0.337 |
| 9* | 0.470 | 0.479 |
| 10* | 0.446 | 0.429 |

*Images of the filaments are not shown.

Supplementary Table S3: **Fluorescence decay times of the donor phiYFP, FRET efficiencies (*E*) and donor-acceptor distances (*r*) in MSR-1 strains, together with their standard deviations (SD) as well as number of bacteria and cultures and statistical analysis.**

| **Constructs** | **$\tau_D$ ± SD [ns]** ♯ | **$\tau_{DA}$ ± SD [ns]** ♯ | **Statistical analysis** § | ***E* ± SD [%]** | ***r* ± SD [nm]** | **Bacteria** | **Indepen-dent cultures** |
|---|---|---|---|---|---|---|---|
| MamJ-phiYFP | 2.94 ± 0.07 (1.00) | | n.s. | | | 9 | 1 |
| phiYFP + TagRFP657-MamK | 2.97 ± 0.08 (1.00) | | *** | | | 34 | 3 |
| MamJ-phiYFP + TagRFP657-MamK (before mechanical treatment) | 2.94 (0.27± 0.10) | 0.56 ± 0.13 (0.73 ± 0.10) | *** | 81 ± 4 | 3.6 ± 0.2 | 72 | 7 |
| MamJ-phiYFP + TagRFP657-MamK (after mechanical treatment) | 2.94 (0.19 ± 0.10) | 0.46 ± 0.17 (0.81 ± 0.10) | n.s. | 84 ± 5 | 3.4 ± 0.2 | 45 | 5 |

♯ Mean amplitudes $\alpha_i$ ± SD of the respective decay time components $\tau_i$ of single- and bi-exponential decay analyses are presented in parentheses.

§ Due to non-normality (D'Agostino and Pearson omnibus normality test, P > 0.05) data were analyzed using a Kruskal-Wallis test followed by Dunn's multiple comparison tests as indicated. n.s.: non-significant, *** P < 0.001.

Supplementary Table S4: **Determined area of the MamK filament before and after applying a magnetic torque.**

| Filament | $I_F$ before magnetic treatment | $I_F$ after magnetic treatment |
|---|---|---|
| Figure 3c and d | 0.564 | 0.483 |
| Supplementary Figure S3a and d | 0.472 | 0.475 |
| Supplementary Figure S4a and d | 0.471 | 0.358 |
| 4* | 0.519 | 0.262 |
| 5* | 0.390 | 0.358 |
| 6* | 0.317 | 0.270 |
| 7* | 0.472 | 0.318 |
| 8* | 0.576 | 0.403 |
| 9* | 0.410 | 0.338 |
| 10* | 0.380 | 0.341 |

*Images of the filaments are not shown.

Supplementary Table S5: **Bacterial strains generated and used in this study.**

| Strain* | Genotype or characteristics | Reference or source |
|---|---|---|
| *M. gryphiswaldense* | | |
| MSR WT | Wild-type MSR-1 R3/S1 (Rif$_R$, Sm$_R$). | 5 |
| Δ*mamJ* | Δ*mamJ* | 6 |
| Δ*mamK* | Δ*mamK* | 7 |
| Δ*mamJK* | Δ*mamJK* | 4 |
| MamJ-phiYFP | MSR WT, conjugated with pMT092, Km$_R$ | This work |
| TagRFP657-MamK | MSR WT, conjugated with pMT094, Km$_R$ | This work |
| MamJ-<br>phiYFP + TagRFP657-<br>MamK | MSR WT, conjugated with pMT095, Km$_R$ | This work |
| phiYFP + TagRFP657-<br>MamK | MSR WT, conjugated with pMT107, Km$_R$ | This work |
| phiYFP | MSR WT, conjugated with pMT108, Km$_R$ | This work |
| Δ*mamJ* + MamJ-phiYFP | Δ*mamJ*, conjugated with pMT092, Km$_R$ | This work |
| Δ*mamK* + TagRFP657-<br>MamK | Δ*mamK*, conjugated with pMT094, Km$_R$ | This work |
| Δ*mamJK* +<br>MamJ-<br>phiYFP + TagRFP657-<br>MamK | Δ*mamJK*, conjugated with pMT095, Km$_R$ | This work |
| *E. coli* | | |
| DH5α | Host for cloning.<br>F- φ80*lac*ZΔM15 Δ(*lac*ZYA-*arg*F) U169 *rec*A1 *end*A1 *hsd*R17 (rK-, mK+) *pho*A *sup*E44 λ-*thi*-1 *gyr*A96 *rel*A1 | Invitrogen |
| WM3064 | Host for cloning and conjugation.<br>*thrB1004 pro thi rpsL hsdS lac*ZΔM15 *RP4-1360* Δ(*araBAD*)*567* Δ*dapA1341*::[*erm pir* (*wt*)] | W. Metcalf,<br>(unpublished) |

Supplementary Table S6: **Plasmids generated and used in this study.**

| Plasmid | Relevant characteristics | Reference or source |
|---|---|---|
| pBBR1-MCS2 | Replicative backbone vector for *in trans* gene expression in MSR. *ori*T, mob, $Km^R$ | [8] |
| pMT080 | pBBR1-MCS2 based vector, $Km^R$, $P_{mamAB}$-*α-helix* (linker) | [4] |
| pMT082 | pBBR1-MCS2 based vector, $Km^R$, $P_{mamAB}$-*mamJ-dendra2* | [4] |
| pMT092 | pMT082 derivative, $P_{mamAB}$-*mamJ-phiYFP* | This work |
| pMT093 | pMT080 derivative, $P_{mamAB}$-*RFP657*-linker | This work |
| pMT094 | pMT093 derivative, $P_{mamAB}$-*TagRFP657-mamK* | This work |
| pMT095 | pMT092 derivative, $P_{mamAB}$-*mamJ-phiYFP_TagRFP657-mamK* | This work |
| pMT107 | pMT095 derivative, $P_{mamAB}$-*phiYFP_TagRFP657-mamK* | This work |
| pMT108 | pMT107 derivative, $P_{mamAB}$-*phiYFP* | This work |

Supplementary Table S7: **Oligonucleotides used in this work.**

| Name | Sequence 5'→3' | Remarks |
|---|---|---|
| oMTN045 | agacta**GGATCC**TCACTGACCGGAAACGTCACCAAGC | Overhang, *Bam*HI |
| oMTN292 | agacta**AAGCTT**ATGAGCAGCGGCGCCCTGCT | Overhang, *Hind*III |
| 0MTN293 | agacta**GGATCC**TCACAGGTAGGTCTTGCGGCAATCC | Overhang, *Bam*HI |
| oMTN294 | agacta**CATATG**AGCGAGCTGATTACCGAGAACATGC | Overhang, *Nde*I |
| oMTN295 | agacta**GAATTC**ATTCAGCTTGTGCCCCAGTTTGCTAGG | Overhang, *Eco*RI |
| oMTN296 | agacta**AAGCTT**ATGAGTGAAGGTGAAGGCCAGGCC | Overhang, *Hind*III |
| oMTN297 | agacta**GGATCC**ctgacccttgaattaaggacaacagcgATGAGCGAGCTGATTACCGAGAACATGC | Overhang, *Bam*HI, Lowercases: *mamC* intergenic region |
| oMTN298 | agacta**CTCGAG**TCACTGACCGGAAACGTCACCAAGCTG | Overhang, *Xho*I |
| oMTN353 | agacta**CATATG**AGCAGCGGCGCCCTGCT | Overhang, *Nde*I |
| oMTN355 | agacta**CTCGAG**TCACTGACCGGAAACGTCACCAAGC | Overhang, *Xho*I |

## References


1. Becker, W. Fluorescence lifetime imaging--techniques and applications. *J. Microsc.* **247,** 119–36 (2012).
2. Jahn, K., Buschmann, V. & Hille, C. Simultaneous Fluorescence and Phosphorescence Lifetime Imaging Microscopy in Living Cells. *Sci. Rep.* **5,** 14334 (2015).
3. Wahl, M. & Orthaus-Müller, S. Time tagged time-resolved fluorescence data collection in life science. *Tech. note, PicoQuant GmbH* (2014).
4. Toro-Nahuelpan, M. *et al.* Segregation of prokaryotic magnetosomes organelles is driven by treadmilling of a dynamic actin-like MamK filament. *BMC Biol.* **14,** 88 (2016).
5. Schultheiss, D. & Schüler, D. Development of a genetic system for Magnetospirillum gryphiswaldense. *Arch. Microbiol.* **179,** 89–94 (2003).
6. Scheffel, A. *et al.* An acidic protein aligns magnetosomes along a filamentous structure in magnetotactic bacteria. *Nature* **440,** 110–114 (2006).
7. Katzmann, E., Scheffel, A., Gruska, M., Plitzko, J. M. & Schüler, D. Loss of the actin-like protein MamK has pleiotropic effects on magnetosome formation and chain assembly in Magnetospirillum gryphiswaldense. *Mol. Microbiol.* **77,** 208–224 (2010).
8. Kovach, M. E., Phillips, R. W., Elzer, P. H., Roop, R. M. & Peterson, K. M. pBBR1MCS: a broad-host-range cloning vector. *Biotechniques* **16,** 800–2 (1994).
